# Applicability of portable retinal cameras and telemedicine as facilitating tools in screening diabetic retinopathy in the COVID-19 pandemic scenario

**DOI:** 10.5935/0004-2749.2021-0498

**Published:** 2022-10-19

**Authors:** Francyne Veiga Reis Cyrino, Suellen Ferronato, Samilla Augusto Vieira de Araujo, Vanessa Giachetto, Layse Dorneles Saud

**Affiliations:** 1 Hospital das Clínicas, Faculdade de Medicina de Ribeirão Preto, Universidade de São Paulo, Ribeirão Preto, SP, Brazil; 2 Centro Avançado de Oftalmologia, Universidade de Ribeirão Preto, Ribeirão Preto, SP, Brazil

**Keywords:** Diabetic retinopathy/diagnosis, Covid-19, Retina/diagnostic imaging, Ophthalmology/instrumentation, Ophthalmoscopes, Point-of-care systems, Telemedicine/methods, Retinopatia diabética/diagnóstico, Covid-19, Re tina/diagnóstico por imagem, Oftalmologia/instrumentação, Oftalmoscópicos, Sistemas automatizados de assistência junto ao leito, Telemedicina/métodos

## Abstract

**Purpose:**

Diabetes mellitus is a leading cause of impaired vision. The objective of this study was to evaluate the feasibility of use of portable retinograph and remote analysis of images along with a virtual questionnaire for screening for diabetic retinopathy in basic health units in the city of Ribeirão Preto/SP during the Covid-19 pandemic.

**Methods:**

Standard Covid-19 protocol was followed during the screening. Blood pressure and capillary blood glucose were measured. Demographic and social data were collected through a standardized online questionnaire via smartphone. After pupillary dilation, fundal images were obtained with portable retinographs by trained ophthalmology residents. Two standardized 45° images were acquired: one posterior segment and another nasal to the optic nerve. Diabetic retinopathy was classified according to the Early Treatment Diabetic Retinopathy Study.

**Results:**

A total of 350 patients (64% female; 45% aged 55-70 years; 55% Caucasian) were evaluated. For 40.5% of patients, the campaign was the first opportunity for retinal evaluation; 47.56% had diabetes mellitus for >10 years. On repeat analysis of images stored in a cloud-based repository by retinal specialist, a 7.8% difference was observed in the Early Treatment Diabetic Retinopathy Study diabetic retinopathy classification, compared to the screening findings. Mild diabetic retinopathy was observed in 12.23%, moderate diabetic retinopathy in 6.31%, and proliferative diabetic retinopathy in 2.58% patients. Macular edema was present in 4.58% patients. Diabetic retinopathy was not detected in 72.78% patients.

**Conclusion:**

Use of portable retinographs together with telemedicine can provide efficient alternative to traditional methods for screening and diagnosis of diabetic retinopathy.

## Abstract (pt)

**Objetivo:**

A diabetes mellitus é considerada uma epidemia global e causa de baixa visual em países em desenvolvimento. Este estudo foi realizado com o objetivo de avaliar a viabilidade do retinógrafo portátil e análise remota de imagens associada a questionário virtual para o rastreio de retinopatia diabética em Unidades Básicas de Saúde da cidade de Ribeirão Preto/SP durante a pandemia de Covid-19.

**Métodos:**

Trezentos e sessenta pacientes compareceram a campanha. O acolhimento foi realizado na Unidade Básica de Saúde pela equipe de enfermagem, respeitando medidas de prevenção do Covid-19 Os realizou-se aferição da pressão arterial e glicemia capilar seguida de dilatação. Dados demográficos e sociais foram coletados através de questionário on-line padronizado via smartphone e realizou-se a triagem da retinopatia diabética através da obtenção de imagens com retinógrafos portáteis realizados por residentes de oftalmologia previamente treinados, com a aquisição de 2 imagens padronizadas de 45º: uma do segmento posterior e outra nasal ao nervo óptico.

**Resultados:**

Trezentos e sessenta pacientes foram atendidos durante a campanha. Dez pacientes (1,02%) foram excluídos devido à opacidade de meios e impossibilidade de obtenção de imagens de fundo de olho. Foram avaliados 350 pacientes, 64% do sexo feminino, 45% entre 55 e 70 anos e 55% brancos. A Campanha foi a primeira avaliação de retina para 40,5% dos pacientes e 47,56% apresentavam diabetes mellitus há mais de 10 anos. Na análise comparativa da classificação da retinopatia diabética segundo *Early Treatment Diabetic Retinopathy Study* (triagem X Nuvem) observou-se uma diferença de 7,8% nos resultados. Retinopatia diabética leve foi observada em 12,23%, moderada em 6,31%, proliferativa em 2,58%; edema macular presente em 4,58% e ausência de retinopatia diabética em 72,78% dos pacientes.

**Conclusão:**

A utilização de retinógrafos portáteis juntamente a telemedicina, para o rastreamento da retinopatia diabética pode ser considerada uma alternativa eficiente para triagem e diagnóstico da retinopatia diabética dentro ou fora do cenário pandêmico, auxiliando na prevenção de perda visual pelo diabetes.

## INTRODUCTION

Diabetes Mellitus (DM) has a great impact on the global health system. The incidence of DM has shown an exponential growth over the last few decades, with approximately 495 million diabetic patients worldwide^(1)^. In Brazil, the estimated case load of patients with DM is approximately 24 million; in addition, Brazil ranks third in the world in terms of expenditure incurred on complications secondary to DM^(2)^. The microvascular and macrovascular changes caused by DM are responsible for multi-system involvement, including vision loss due to diabetic retinopathy (DR). According to data released by the Brazilian Council of Ophthalmology (CBO) in 2019, DR is the leading cause of irreversible blindness among Brazilian population in the productive age-group^(3)^.

A recent study by Virk et al. demonstrated a strong association between the number of appointments missed for screening for DR and the number of patients who develop DR^(4)^. Missing 5 consecutive appointments was associated with 4%-15% higher risk of retinopathy, while missing 10 appointments increased the risk by 20%^(4)^.

The social-distancing norms and restricted access to healthcare services during the Covid-19 pandemic posed a barrier to health care delivery, especially for patients at a high risk of complications, such as diabetes patients. This necessitated the implementation of new strategies for screening of these patients for DR to prevent vision loss, with the aim to increase accessibility, accuracy, and efficiency.

The aim of the study is to present the findings of the use of a handheld retinal camera (smartphone-based handheld device, Eyer, Phelcon Technologies, São Carlos, Brazil) for screening of patients for DR during the Ribeirão Preto Campaign of Diabetes in five Basic Health Units in the city.

## METHODS

### Study design and patient selection

The study was carried out at five Basic Health Units (BHU) of Ribeirão Preto city, São Paulo, Brazil, on October 26^th^ and 27^th^, and November 4^th^, 9^th^, and 11^th^, 2020. BHUs were selected by the Ribeirão Preto Health Department (HD-RP) based on the availability of physical space to avoid crowding during screening. The entire study and evaluation format were carried out according to the protocols established by the Ministry of Health.

The study was conducted in accordance with the principles of the Declaration of Helsinki, and the protocol was approved by the Ethics Committee of the University of Ribeirão Preto - UNAERP.

Eight hundred diabetic patients were telephonically contacted by the BHU of Ribeirão Preto/SP and invited to participate in DR screening at pre-scheduled times, with a maximum of 4 patients per hour, to reduce the waiting time at the unit and minimize the risk of Covid-19 transmission. One hundred and eighty assessments were made available per day (20 patients per hour) from 8 am to 5 pm. Out of the 800 patients invited for screening, only 360 patients attended the 5-day campaign.

The patients were received at the BHU by the nursing staff followed the standard Covid-19 protocol [maintenance of social distance (1 meter), use of alcohol gel for hand hygiene, masks. All patients underwent measurement of blood pressure and capillary blood glucose level at reception.

**Figure 1:**
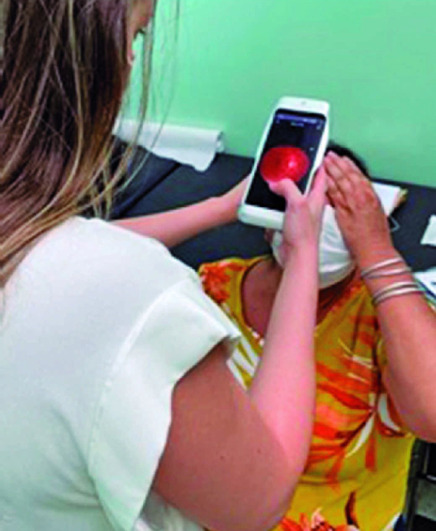
Ophthalmology resident acquiring fundal images using EYER portable retinal camera.

In 2020, the annual project of the Diabetes Campaign in Ribeirão Preto/SP was conducted at the BHUs, and DR screening was performed by obtaining images (retinography) obtained using four portable retinal cameras (EYER, Phelcon Technologies, BR). The images were obtained by five Ophthalmology residents of the Advanced Center of Ophthalmology at the University of Ribeirão Preto (CAO -UNAERP) who were previously trained to acquire the best image during screening (focus and centralization), supervised and coordinated by the retinal specialist (FVRC) (Figure 1). The acquired images were uploaded to a cloud-based repository for further analysis and confirmation of diagnosis and/or classification of DR by the retinal specialist.

### Preparation for acquisition of images and virtual questionnaire

Prior to ocular imaging, all patients signed an informed consent form and completed a standardized online questionnaire (containing demographic and social data) via smartphone. The online questionnaire was prepared on a platform called SurveyMonkey.com (www.surveymonkey.com), in its paid version, which allows simultaneous access to the questionnaire by several smartphones, allowing real-time acquisition of answers and streamlining the work of resident physicians and trainees in obtaining the necessary information in the shortest possible time.

Subsequently, all patients were administered 1% tropicamide eye drops (one drop in each eye) for pupillary dilation 15 minutes prior to fundus examination. The dilation was performed despite the fact that the mobile image capture device was non-mydriatic to avoid any problems during the further evaluation of the images.

### Obtaining and analyzing images

**Figure 2:**
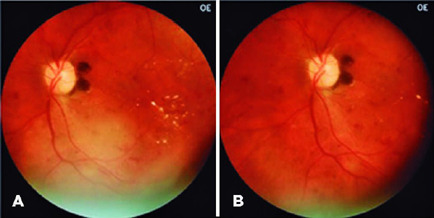
Images acquired based on standardization. A) posterior segment. B) Nasal to the optic nerve.

Four non-contact fundus imaging handheld devices (portable retinal camera EYER, Phelcon Technologies, BR) attached to a Samsung Galaxy S10 smartphone and an Android 11 system were used in order to reduce physical proximity to the patient. In all patients, two 45º images were standardized: from the posterior segment, positioning the optic nerve nasally, and nasal to the optic nerve, positioning it temporally as described by Malerbi et al.^(5)^ (Figure 2). In case of doubt about any of the images, frames of other quadrants could also be acquired.

After evaluation of each patient, a second online questionnaire was answered about the presence or absence of DR, DR classification, presence or absence of clinically significant macular edema, and the treatment indicated.

Afterwards, all images were automatically stored in a cloud-based repository hosted by PHELCON Technologies (Eyer Cloud) for further evaluation and made available for access at any time. Despite the supervision of the retinal specialist (FVRC) at the BHU, another more careful analysis of all images stored in the Cloud was performed by the specialist and the findings were compared to the findings obtained during the screening.

After the evaluation, all patients received an orientation booklet containing notes on capillary glycemia, blood pressure, presence or absence of DR, classification, indicated treatment, and clinical follow-up. A copy was delivered to HDRP for scheduling the indicated/prioritized treatment.

## RESULTS

**Figure 3:**
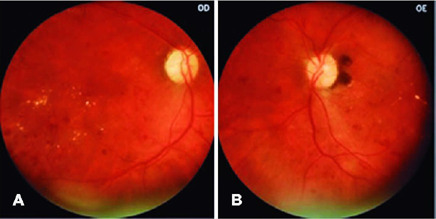
Representative case of severe non-proliferative diabetic retinopathy and macular edema (right and left eye, respectively).

**Figure 4:**
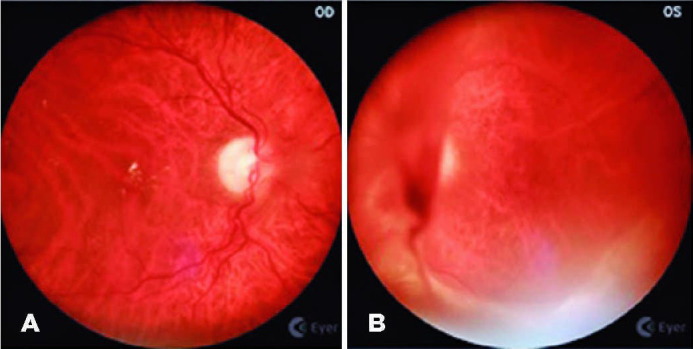
(A) High risk proliferative diabetic retinopathy with active neovascularization at the disc and macular edema in the right eye. (B) Vitreous hemorrhage from active neovessels at the disc and fibrovascular proliferation at inferior arcade on the left eye.

**Figure 5:**
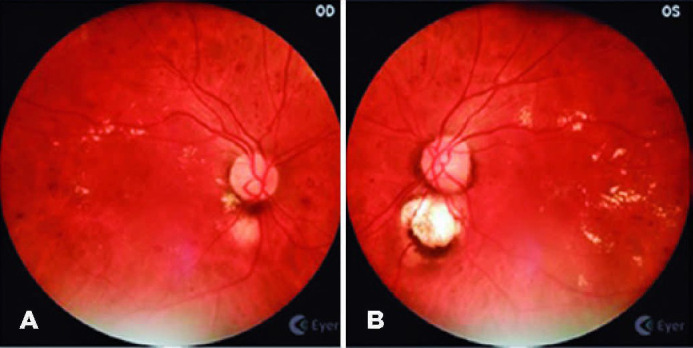
Representative case of severe non-proliferative diabetic retinopathy with macular edema (right and left eye, respectively) and coloboma in the inferior part of the disc, in both eyes.

Out of the 360 patients who attended the screening, 10 patients (1,02%) were excluded due to media opacities and inability to obtain fundus images. Thus, a total of 350 patients were included in this study. The standardized online questionnaire and non-contact fundus images obtained with handheld devices were acquired for analysis; in addition, a subsequent reanalysis of the images stored in the cloud was performed by the retinal specialist (FVRC) (Figures 3, 4, and 5).

In this study, 64% patients were female; the age distribution in our cohort was as follows: 45% aged between 55 and 70 years; 28% aged >75 years; 21% aged between 40 and 55 years; 6% aged <40 years (median age: 59.2 years); 55% of patients were Caucasian. For 40.5% of patients, the campaign was the first opportunity to undergo retinal evaluation and they had never been referred to an ophthalmologist before. The majority of patients (81.74%) were receiving follow-up care for diabetes at BHUs and 47.56% had DM for >10 years.

On repeat analysis of images stored in the Cloud, a 7.8% difference in the ETDRS DR classification was observed compared to the findings reported at screening. The images were reclassified, always using ETDRS as the standard methodology. The findings related to the presence and severity of DR are presented in table 1.

**Table 1:** Distribution of diabetic retinopathy and its severity in the study population

Diabetic retinopathy severity level	Distribution (%)	Lesions
Absent	72.78	No alterations
Mild NPDR	12.23	At least one hemorrhage or microaneurysm
Moderate NPDR	6.31	Four or more hemorrhages in only one hemi-field^a^
Severe NPDR	2.58	Any of the following: - Four or more hemorrhages in the superior and inferior hemi-fields - Venous beading - Intraretinal microvascular abnormalities (IRMA)
Proliferative diabetic retinopathy	4.58	Any of the following: - Active neovessels - Vitreous hemorrhage

NPDR= non-proliferative diabetic retinopathy.

a = Superior and inferior hemi-fields separated by the line passing through the center of the macula and the optic disc.

Results of capillary blood glucose, with or without fasting at the time of evaluation are shown in table 2. Sixty-two percent patients in our cohort were not aware of the ideal mean blood glucose level, 53% patients considered having the disease under control, and 29% were not aware of the complications of DM.

**Table 2:** Results of assessment of capillary blood glucose level

Glycemic values on the day of the screening	Fasting blood glucose (50 patients)	Glycemic values on the day of the screening	Random blood glucose (300 patients)
≥100 mg/dl	10	≥140 mg/dl	99
101-140 mg/dl	10	141-180 mg/dl	40
141-180 mg/dl	19	181-200 mg/dl	100
181-200 mg/dl	01	>300 mg/dl	60
>200 mg/dl	10	>500 mg/dl	01
Total patients	**350 patients**		

Patients who required laser therapy, antiangiogenic treatment, surgery, or follow-up were referred to the Health Department of Ribeirão Preto for scheduling.

## DISCUSSION

Brazil has the fifth largest population of diabetics in the world and DR is one of the main preventable causes of blindness. The Covid-19 pandemic offered us an opportunity to improve the screening strategy for this disease. Ophthalmologists are among the groups of physicians that are most at risk of contracting the virus during care, due to their physical proximity to the patient^(5,6)^. Therefore, use of mobile devices, such as smartphones and others portable retinal cameras, can help improve the accessibility and effectiveness of screening, early diagnosis, and treatment^(7)^.

In a recent systematic review, Kashim et al. identified several factors that act as barriers to screening for DR, even in non-pandemic periods. They identified lower socioeconomic level, younger age, and lack of ready access to information about disease and its complications as risk factors for non-attendance to consultations and non-adherence to treatment^(8)^. In addition, long waiting times on the day of care, irregular interval between appointments, and discomfort associated with administration of mydriatic eye drops were identified as important reasons for not attending consultations regularly^(8)^.

Another important issue is the lack of knowledge about their disease such as ideal glycemic control and potential complications of DM. In our study, approximately 62% of patients were not aware of the ideal blood glucose level, 53% considered having the disease under control, and 29% were not aware about the complications of DM. Kashim et. al. also identified the lack of information as an important reason for lack of engagement, treatment, and attending appointments and/or undergoing investigations^(9)^.

Virk et al. demonstrated an association between missed appointments and progression of DR^(4)^. According to a recent study by Vujosevic et al., access to quality care for diabetic patients and early referral for ophthalmological evaluation are important factors for the prevention of DR and blindness^(9)^. In our study, 45% of the patients had never been referred to an ophthalmologist which shows the importance of education and prevention in diabetes.

Telemedicine and artificial intelligence technologies are increasingly being leveraged to facilitate medical care, especially after the onset of the Covid-19 pandemic. In the context of eye diseases, studies have demonstrated the successful use of telemedicine for the screening of DR, and better monitoring of the disease and its complications within the scope of the Basic Heath Unit^(10,11,12)^.

In the present study, we observed that a healthcare professional well-trained in the use of non-contact portable retinal cameras is capable of obtaining good quality images which can then be analyzed by an expert ophthalmologist who has web-based remote access to these images. These images appear immediately on the device, which facilitates real-time analysis of the quality of images, correct angle, and media opacity, and the need for new photos. However, it is very important to train these professionals to acquire the best images so that these can be remotely analyzed later.

In the current health system in Brazil, there is a long period between the first evaluation by general ophthalmologist and the tertiary service (retinal specialist), if treatment is needed. With this strategy, a retinal specialist is able to access a diverse range of images from several patients irrespective of their geographical location in a much shorter period of time^(4,13)^.

Portable retinal cameras are a low-cost and highly efficient alternative that can promote screening, follow-up, and diagnosis of DR irrespective of the pandemic scenario. By remote analysis of the acquired fundus photographs using telemedicine technologies, it is possible to identify and correctly classify DR. In addition, this strategy is able to reach a larger population in less time, enabling faster access to specialist treatment and preventing visual loss.

It is pertinent to mention here that use of portable retinal cameras and/or telemedicine and even artificial intelligence are not a replacement for the current screening system. On the contrary, these technologies provide a new way to increase DR diagnosis and to assist the ophthalmologist in this process; such an approach can promote greater flexibility with respect to the referral of cases that require treatment to the retinal specialist.

Screening for DR using portable retinal cameras and use of standardized online questionnaires proved to be an effective, inexpensive, and convenient alternative to the traditional forms of screening.

Likewise, our findings highlight the need for more atten tion towards screening for DR (requesting eye fundus exams) at BHU level. Moreover, healthcare professionals working at BHU should educate diabetes patients regarding the systemic and ocular complications of diabetes, ideal blood glucose level and provide appropriate dietary counseling.

Furthermore, it is extremely important to create counter-referral protocols for patients with DR/macular edema in order to avoid treatment delay and reduce the risk of blindness.
